# α-Lead tellurite from single-crystal data

**DOI:** 10.1107/S1600536808003267

**Published:** 2008-02-06

**Authors:** Valery E. Zavodnik, Sergey A. Ivanov, Adam I. Stash

**Affiliations:** a Karpov Institute of Physical Chemistry, 10 Vorontsovo Pole, 105064 Moscow, Russian Federation; b Materials Chemistry, Uppsala University, Box 538, SE-75121, Uppsala, Sweden

## Abstract

The crystal structure of the title compound, α-PbTeO_3_ (PTO), has been reported previously by Mariolacos [*Anz. Oesterr. Akad. Wiss. Math. Naturwiss. Kl*. (1969), **106**, 128–130], refined on powder data. The current determination at room temperature from data obtained from single crystals grown by the Czochralski method shows a significant improvement in the precision of the geometric parameters when all atoms have been refined anisotropically. The selection of a centrosymmetric (*C*2/*c*) structure model was confirmed by the second harmonic generation test. The asymmetric unit contains three formula units. The structure of PTO is built up of three types of distorted [PbO_*x*_] polyhedra (*x* = 7 and 9) which share their O atoms with TeO_3_ pyramidal units. These main anionic polyhedra are responsible for establishing the two types of tunnel required for the stereochemical activity of the lone pairs of the Pb^2+^ and Te^4+^ cations.

## Related literature

Single crystals of PTO were grown by the Czochralski technique (Kosse, Politova, Bush *et al.*, 1983 ▶). For the temperature dependence of the physical properties of PTO, see: Kosse, Politova, Astafiev *et al.* (1983 ▶). For the polymorphism of PTO, see: Tananaeva *et al.* (1977 ▶), Robertson *et al.* (1976 ▶), Young (1979 ▶). Several different polymorphs were previously described as monoclinic (Mariolacos, 1969 ▶), triclinic (Williams, 1979 ▶), ortho­rhom­bic (Spiridonov & Tananaeva, 1982 ▶), tetra­gonal (Sciau *et al.*, 1986 ▶) and cubic (Gaitan *et al.*, 1987 ▶). For related literature, see: Brown (1974 ▶); Galy *et al.* (1975 ▶); Gillespie (1972 ▶); Tananaeva & Novoselova (1977 ▶).

## Experimental

### 

#### Crystal data

PbTeO_3_
*M* *_r_* = 382.79
Monoclinic, 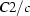

*a* = 26.555 (5) Å
*b* = 4.593 (1) Å
*c* = 17.958 (4) Å
β = 106.97 (3)°
*V* = 2094.9 (7) Å^3^
*Z* = 24
Mo *K*α radiation
μ = 56.32 mm^−1^
*T* = 295 (2) K
0.14 × 0.04 × 0.02 mm

#### Data collection

Enraf–Nonius CAD-4 diffractometer
Absorption correction: refined from Δ*F* (Walker & Stuart, 1983 ▶) *T*_min_ = 0.234, *T*_max_ = 0.695 (expected range = 0.109–0.324)
3717 measured reflections
3608 independent reflections
1676 reflections with *I* > 2σ(*I*)
*R*_int_ = 0.054
3 standard reflections frequency: 60 min intensity decay: none

#### Refinement

*R*[*F* ^2^ > 2σ(*F* ^2^)] = 0.026
*wR*(*F* ^2^) = 0.063
*S* = 0.77
3608 reflections
137 parameters
Δρ_max_ = 2.31 e Å^−3^
Δρ_min_ = −2.06 e Å^−3^

### 

Data collection: *CAD-4-PC* (Enraf–Nonius, 1993 ▶); cell refinement: *CAD-4-PC*; data reduction: *CAD-4-PC*; program(s) used to solve structure: *SHELXS97* (Sheldrick, 2008 ▶); program(s) used to refine structure: *SHELXL97* (Sheldrick, 2008 ▶); molecular graphics: *DIAMOND* (Brandenburg, 2005 ▶); software used to prepare material for publication: *CIFTAB97* (Sheldrick, 2008 ▶).

## Supplementary Material

Crystal structure: contains datablocks I, global. DOI: 10.1107/S1600536808003267/fi2057sup1.cif

Structure factors: contains datablocks I. DOI: 10.1107/S1600536808003267/fi2057Isup2.hkl

Additional supplementary materials:  crystallographic information; 3D view; checkCIF report

## Figures and Tables

**Fig. 1.**
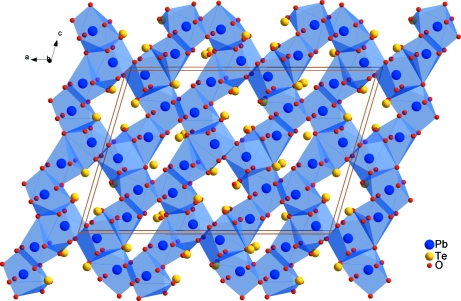
Polyhedral representation of the structure of α-PbTeO3.

**Fig. 2.**
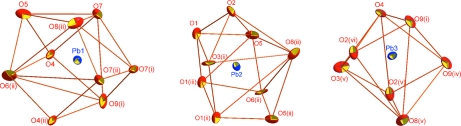
Coordination polyhedra of the different Pb cations. Displacement ellipsoids are drawn at the 50% probability level. The labeling scheme for symmetry-related atoms is the following: (i) 0.5 - x,1/2 + y,1.5 - z˛ (ii) x,1 + y,z˛ (iii) -x,-y,1 - z˛ (iv) 0.5 - x,-1/2 + y,1.5 - z˛ (v) x,-1 - y,1/2 + z˛ (vi) x,-y,1/2 + z.

## supplementary crystallographic information

### Comment

Crystals with the Pb^2+^ and Te^4+^ cations having stereochemically active lone-pairs are very attractive materials for ferroelectric and non-linear optical applications. The knowledge of the crystal structures of these compounds should provide important information about the unusual mechanism of formation of their polar properties. The investigation of the PbO-TeO_2_ system (Robertson *et al.*, 1976; Young, 1979) has provided evidence of a large number of different phases. The polymorphism, crystal structure and thermodynamic status of PbTeO_3 _(PTO) are not fully established and literature reports give conflicting statements (Tananaeva *et al.*,1977; Robertson *et al.*,1976; Young, 1979). Several different polymorphs have previously been described: monoclinic (Mariolacos, 1969), triclinic (Williams, 1979), tetragonal (Sciau *et al.*, 1986) and cubic (Gaitan *et al.*,1987). It should be mentioned that Spiridonov & Tananaeva (1982) described α-PbTeO_3_ as orthorhombic. The tetragonal phase was shown to be ferroelectric. The phase change from the tetragonal to the monoclinic form at 783 K has been shown to be irreversible (Young, 1979). The present paper deals with the crystal structure determination of α-PTO. This structure can be described in terms of complex irregular Pb^2+^ polyhedra with 7 and 9 apices and separate Te^4+^O_3_ groups (Fig. 1,2). Three kinds of Pb—O distances can be distinguished: three short contacts (2.25–2.53 Å), three longer distances (2.63–2.96 Å) and three abnormally long distances (3.02–3.26 Å). The different Pb polyhedra are connected by face, edge and corner sharing through the Pb—O bonds forming the network with the honeycomb-like chains parallel to *c* axis. The Te^4+^ cations coordinate to three O atoms in a one-sided pyramidal coordination TeO_3_E (E are lone-pair electrons). The Te—O distances are in the range 1.85–1.90 Å. The O—Te—O angles are close to 100°. The next-nearest anions are located at distances greater than 2.7 Å. In accordance with Brown (1974) these additional weak contacts are important for the determination of the correct coordination geometry of the Te cations. Depending on the type of Te^4+^O_3_ E units, two types of tunnels are formed running along [010], which represent the required space for the electron lone pairs within the structure. According to Gillespie (1972), Galy *et al.* (1975) the electronic lone pair E is sitting inside these non-bonding regions.

### Experimental

Single crystals of PTO were grown by the Czochralski technique as described earlier (Kosse, Politova, Bush *et al.*, 1983; Kosse, Politova, Astafiev *et al.*, 1983). The chemical composition of tested crystals was confirmed with energy-dispersive spectrometry analysis (LINK AN10000). Second harmonic generation (SHG) measurements showed no positive signals at room temperature which is in accordance with the given space group.

### Refinement

The structure of PTO was solved by the direct method in space group *C*2/*c* where the atomic coordinates of all Pb and Te cations were found. The oxygen atoms were localized by difference Fourier maps.

The very high absorption coefficient (µ=56.32 mm^-1^) and imperfect shape of crystal are the reason why the program *DIFABS* (Walker & Stuart, 1983) was used for absorption correction.

The highest residual electron density peak (2.31 e A^-3^) is located 1.00Å from atom Pb1 and the deepest hole

(-2.06 e A^-3^) is located 1.46Å from atom O4.

### Crystal data

**Table:**

PbTeO_3_	*F*_000_ = 3792
*M**_r_* = 382.79	*D*_x_ = 7.282 Mg m^−^^3^
Monoclinic, *C*2/*c*	Mo *K*α radiation λ = 0.71073 Å
Hall symbol: -C 2yc	Cell parameters from 24 reflections
*a* = 26.555 (5) Å	θ = 12.1–14.5º
*b* = 4.593 (1) Å	µ = 56.32 mm^−^^1^
*c* = 17.958 (4) Å	*T* = 295 (2) K
β = 106.97 (3)º	Needle, colourless
*V* = 2094.9 (7) Å^3^	0.14 × 0.04 × 0.02 mm
*Z* = 24	

### Data collection

**Table:**

Enraf–Nonius CAD-4 diffractometer	*R*_int_ = 0.054
Radiation source: fine-focus sealed tube	θ_max_ = 32.0º
Monochromator: β-filter	θ_min_ = 1.6º
*T* = 293(2) K	*h* = −39→37
ω/2θ scans	*k* = −6→0
Absorption correction: part of the refinement model (Δ*F*)(Walker & Stuart, 1983)	*l* = 0→26
*T*_min_ = 0.234, *T*_max_ = 0.695	3 standard reflections
3717 measured reflections	every 60 min
3608 independent reflections	intensity decay: none
1676 reflections with *I* > 2σ(*I*)	

### Refinement

**Table:**

Refinement on *F*^2^	Secondary atom site location: difference Fourier map
Least-squares matrix: full	*w* = 1/[σ^2^(*F*_o_^2^) + (0.0301*P*)^2^] where *P* = (*F*_o_^2^ + 2*F*_c_^2^)/3
*R*[*F*^2^ > 2σ(*F*^2^)] = 0.026	(Δ/σ)_max_ = 0.001
*wR*(*F*^2^) = 0.063	Δρ_max_ = 2.31 e Å^−^^3^
*S* = 0.77	Δρ_min_ = −2.06 e Å^−^^3^
3608 reflections	Extinction correction: SHELXL97 (Sheldrick, 2008), Fc^*^=kFc[1+0.001xFc^2^λ^3^/sin(2θ)]^-1/4^
137 parameters	Extinction coefficient: 0.000052 (5)
Primary atom site location: structure-invariant direct methods	

### Special details

**Table:**

Geometry. All e.s.d.'s (except the e.s.d. in the dihedral angle between two l.s. planes) are estimated using the full covariance matrix. The cell e.s.d.'s are taken into account individually in the estimation of e.s.d.'s in distances, angles and torsion angles; correlations between e.s.d.'s in cell parameters are only used when they are defined by crystal symmetry. An approximate (isotropic) treatment of cell e.s.d.'s is used for estimating e.s.d.'s involving l.s. planes.
Refinement. Refinement of *F*^2^ against ALL reflections. The weighted *R*-factor *wR* and goodness of fit *S* are based on *F*^2^, conventional *R*-factors *R* are based on *F*, with *F* set to zero for negative *F*^2^. The threshold expression of *F*^2^ > σ(*F*^2^) is used only for calculating *R*-factors(gt) *etc*. and is not relevant to the choice of reflections for refinement. *R*-factors based on *F*^2^ are statistically about twice as large as those based on *F*, and *R*- factors based on ALL data will be even larger.

### Fractional atomic coordinates and isotropic or equivalent isotropic displacement parameters (Å^2^)

**Table:**

	*x*	*y*	*z*	*U*_iso_*/*U*_eq_	
Pb1	0.183652 (15)	0.21366 (10)	0.73592 (2)	0.01749 (9)	
Pb2	0.066493 (14)	0.22395 (12)	0.54345 (2)	0.01847 (9)	
Pb3	0.162024 (14)	−0.24695 (10)	0.913902 (19)	0.01916 (9)	
Te1	0.04734 (2)	−0.23056 (15)	0.36782 (3)	0.01331 (11)	
Te2	0.06941 (2)	−0.25178 (16)	0.70793 (3)	0.01367 (11)	
Te3	0.20758 (3)	−0.30201 (15)	0.59262 (4)	0.01439 (13)	
O1	0.0204 (3)	−0.179 (2)	0.4532 (4)	0.0240 (17)	
O2	0.1172 (3)	−0.156 (2)	0.4259 (5)	0.0285 (19)	
O3	0.0559 (4)	−0.6348 (18)	0.3703 (6)	0.034 (2)	
O4	0.1359 (3)	−0.221 (2)	0.7823 (4)	0.0201 (15)	
O5	0.0952 (4)	−0.1694 (19)	0.6233 (4)	0.028 (2)	
O6	0.0659 (4)	−0.6528 (17)	0.6935 (6)	0.035 (2)	
O7	0.2214 (3)	−0.2120 (18)	0.6999 (4)	0.0180 (14)	
O8	0.1858 (4)	−0.683 (2)	0.6000 (5)	0.033 (2)	
O9	0.2781 (3)	−0.355 (2)	0.6016 (6)	0.034 (2)	

### Atomic displacement parameters (Å^2^)

**Table:**

	*U*^11^	*U*^22^	*U*^33^	*U*^12^	*U*^13^	*U*^23^
Pb1	0.01503 (16)	0.0168 (2)	0.02066 (16)	0.00061 (16)	0.00526 (13)	−0.00209 (16)
Pb2	0.01480 (15)	0.0215 (2)	0.01935 (15)	0.00049 (18)	0.00528 (12)	0.00186 (17)
Pb3	0.02372 (17)	0.02011 (19)	0.01346 (14)	0.00414 (19)	0.00511 (12)	0.00129 (16)
Te1	0.0161 (2)	0.0109 (3)	0.0145 (2)	0.0019 (3)	0.00698 (19)	−0.0003 (2)
Te2	0.0136 (2)	0.0106 (3)	0.0157 (2)	−0.0002 (3)	0.00254 (19)	−0.0021 (3)
Te3	0.0163 (3)	0.0122 (3)	0.0137 (2)	0.0009 (2)	0.0029 (2)	0.0001 (2)
O1	0.022 (4)	0.035 (5)	0.016 (3)	−0.007 (4)	0.008 (3)	−0.003 (3)
O2	0.013 (3)	0.032 (5)	0.037 (4)	0.005 (3)	0.002 (3)	0.007 (4)
O3	0.056 (7)	0.007 (4)	0.040 (5)	0.010 (4)	0.014 (5)	−0.002 (3)
O4	0.017 (3)	0.030 (4)	0.014 (3)	−0.013 (4)	0.006 (2)	−0.004 (3)
O5	0.050 (6)	0.022 (4)	0.014 (3)	0.015 (4)	0.012 (4)	0.010 (3)
O6	0.044 (6)	0.005 (3)	0.057 (6)	−0.004 (4)	0.019 (5)	−0.012 (4)
O7	0.020 (3)	0.014 (3)	0.020 (3)	0.006 (3)	0.007 (3)	0.006 (3)
O8	0.053 (6)	0.021 (4)	0.024 (4)	−0.016 (4)	0.013 (4)	0.000 (3)
O9	0.013 (3)	0.052 (6)	0.039 (5)	0.014 (4)	0.010 (3)	0.000 (4)

### Geometric parameters (Å, °)

**Table:**

Pb1—O7	2.371 (8)	Pb3—O3^v^	2.750 (11)
Pb1—O7^i^	2.471 (7)	Te1—O3	1.870 (8)
Pb1—O8^ii^	2.504 (8)	Te1—O2	1.876 (9)
Pb1—O4	2.628 (8)	Te1—O1	1.888 (7)
Pb2—O5	2.294 (8)	Te2—O6	1.859 (8)
Pb2—O1^iii^	2.334 (8)	Te2—O5	1.878 (8)
Pb2—O1	2.528 (9)	Te2—O4	1.883 (7)
Pb2—O6^ii^	2.758 (10)	Te3—O9	1.848 (8)
Pb3—O2^iv^	2.246 (9)	Te3—O8	1.858 (9)
Pb3—O4	2.263 (6)	Te3—O7	1.899 (7)
Pb3—O9^i^	2.471 (9)		
			
O7—Pb1—O7^i^	77.64 (19)	O6—Te2—O5	95.9 (4)
O7—Pb1—O8^ii^	76.1 (3)	O6—Te2—O4	99.8 (5)
O7^i^—Pb1—O8^ii^	96.7 (3)	O5—Te2—O4	94.1 (4)
O7—Pb1—O4	75.0 (2)	O9—Te3—O8	101.4 (5)
O7^i^—Pb1—O4	118.6 (2)	O9—Te3—O7	92.8 (4)
O8^ii^—Pb1—O4	127.2 (3)	O8—Te3—O7	96.0 (4)
O5—Pb2—O1^iii^	93.7 (3)	Te1—O1—Pb2^iii^	128.4 (4)
O5—Pb2—O1	80.5 (3)	Te1—O1—Pb2	112.6 (4)
O1^iii^—Pb2—O1	69.8 (3)	Pb2^iii^—O1—Pb2	110.2 (3)
O5—Pb2—O6^ii^	69.3 (3)	Te1—O2—Pb3^vi^	124.2 (4)
O1^iii^—Pb2—O6^ii^	72.9 (3)	Te1—O3—Pb3^vii^	108.1 (5)
O1—Pb2—O6^ii^	129.5 (3)	Te2—O4—Pb3	132.5 (3)
O2^iv^—Pb3—O4	92.7 (3)	Te2—O4—Pb1	105.7 (3)
O2^iv^—Pb3—O9^i^	77.7 (3)	Pb3—O4—Pb1	110.0 (3)
O4—Pb3—O9^i^	81.7 (3)	Te2—O5—Pb2	122.1 (4)
O2^iv^—Pb3—O3^v^	70.3 (3)	Te2—O6—Pb2^viii^	109.0 (4)
O4—Pb3—O3^v^	74.8 (3)	Te3—O7—Pb1	119.0 (3)
O9^i^—Pb3—O3^v^	138.7 (3)	Te3—O7—Pb1^ix^	108.2 (3)
O3—Te1—O2	94.2 (4)	Pb1—O7—Pb1^ix^	116.2 (3)
O3—Te1—O1	100.2 (4)	Te3—O8—Pb1^viii^	110.2 (4)
O2—Te1—O1	94.2 (4)	Te3—O9—Pb3^ix^	139.0 (6)

Symmetry codes: (i) −*x*+1/2, *y*+1/2, −*z*+3/2; (ii) *x*, *y*+1, *z*; (iii) −*x*, −*y*, −*z*+1; (iv) *x*, −*y*, *z*+1/2; (v) *x*, −*y*−1, *z*+1/2; (vi) *x*, −*y*, *z*−1/2; (vii) *x*, −*y*−1, *z*−1/2; (viii) *x*, *y*−1, *z*; (ix) −*x*+1/2, *y*−1/2, −*z*+3/2.
